# Comparing the effects of sitting meditation and body scan on working memory in undergraduate healthcare students: a single-blind randomised trial

**DOI:** 10.1186/s40359-026-04923-6

**Published:** 2026-06-04

**Authors:** Weeranan Yaemrattanakul, Kanokwan Manachamni, Jutharat Ruangrattanapong, Worawadee Supajumroon, Worravat Intarapakdee, Kanokwalin Klangkhong, Kanjanaporn Wannasangthong, Nureeda Madhay, Patchanee Kaewkeaw, Korawat Paphatarinan, Lalita Khuna

**Affiliations:** 1 Department of Physical Therapy, Faculty of Medicine, Prince of Songkla University, Songkhla, Thailand; 2 School of Physical Therapy, Faculty of Associated Medical Sciences, Khon Kaen University, Khon Kaen, Thailand

**Keywords:** Sitting meditation, Body scan, Stress, Working memory

## Abstract

**Background:**

Healthcare undergraduate students often face high cognitive and emotional stress, negatively affecting their working memory and well-being. Mindfulness-based practices, such as sitting meditation and body scan, are proposed to enhance cognitive function and reduce stress in this population. This study compared the effects of brief sitting meditation and body scan on working memory, mindfulness, and stress among undergraduate healthcare students.

**Methods:**

In this assessor-blind randomised trial conducted at a tertiary university in southern Thailand, 56 healthcare undergraduates (aged 18–25) were randomised to either a sitting meditation group (*n* = 28) or a body scan group (*n* = 28). Fifty-four participants completed the study and were included in the final analysis (*n* = 27 per group). Participants practised their assigned mindfulness exercise via audio recordings for 10 min daily, five days a week, for four weeks. Outcomes were measured pre- and post-intervention using the Automated Operation Span Task for working memory, Mindful Attention Awareness Scale for mindfulness, and Thai Perceived Stress Scale-10 for stress.

**Results:**

Both groups showed significant changes in working memory and mindfulness over time. No statistically significant differences were observed between the two interventions. Stress scores changed slightly over time; however, no significant difference was observed between the sitting meditation and body scan groups. Overall, no significant between-group differences were found in any outcome.

**Conclusions:**

Both sitting meditation and body scan were associated with changes in working memory and mindfulness over time among undergraduate healthcare students. However, no statistically significant differences were observed between the groups.

## Background

Working memory (WM) is a core component of executive function, allowing individuals to temporarily retain and manipulate information for complex cognitive tasks, such as comprehension, reasoning, and decision-making [1, 2]. Its importance is especially evident in academic contexts, where it is a stronger predictor of scholastic achievement than general intelligence [3, 4]. For students in health science disciplines, demands on WM increase because they must process extensive theoretical knowledge, manage emotionally charged patient interactions, and make high-stakes clinical decisions, all under significant time and performance pressures [5, 6]. These cognitive loads are often increased by persistent stressors, such as heavy workload, clinical duties, institutional expectations, and limited opportunities for recovery, which affect WM by disrupting the prefrontal cortex function through elevated cortisol levels. Ultimately, these factors may hinder both academic achievement and clinical performance [2, 7].

Mindfulness-based interventions (MBIs), including mindfulness-based stress reduction (MBSR) and mindfulness-based cognitive therapy (MBCT), have gained traction in educational settings as strategies to support cognitive performance and mental health. These standardised programs typically comprise weekly 1.5 to 2-hour group sessions, daily home practice of approximately 45 min for six days per week, and a full-day silent retreat in weeks 6 or 7, spanning a total duration of 8 weeks. MBSR incorporates sitting meditation, body scan, and Hatha yoga, whereas MBCT includes sitting meditation and body scan but replaces the Hatha yoga component with cognitive behavioural therapy elements [8, 9]. MBI programs have consistently demonstrated cognitive and emotional benefits in undergraduate healthcare students, including enhanced WM capacity, greater mindfulness, and reduced stress [10, 11]. Meta-analyses have further reported small-to-moderate improvements in WM accuracy and cognitive control following full-length MBIs [12, 13]. Neuroimaging studies suggest that these benefits may be mediated by neuroplastic changes, particularly in the prefrontal cortex, insula, anterior cingulate cortex, and hippocampus—regions involved in self-regulation and memory consolidation [14–16].

Despite these benefits, the substantial time commitment of an 8-week MBI limits its feasibility for undergraduate healthcare students balancing intensive academic workloads and restricted resources [17]. These barriers are particularly salient for younger populations, where travel requirements and program costs further constrain participation. Consequently, shorter or brief MBIs have been developed to improve accessibility and engagement while preserving the core mindfulness principles [13, 18]. Evidence indicates that such adaptations can produce meaningful improvements in WM, mindfulness, and stress reduction [19–21], supporting their feasibility in undergraduate healthcare settings.

However, MBIs typically operate as multicomponent “packages,” making it challenging to isolate the specific practices driving cognitive and emotional gains [22, 23]. Dismantling studies have begun to address this limitation by examining individual mindfulness techniques [24, 25]. For example, Quach et al. [26] compared mindfulness meditation and Hatha yoga in adolescents, finding that a brief, four-week meditation program significantly enhanced WM capacity, whereas Hatha yoga did not—highlighting the potential of short-format, focused mindfulness practices for targeted cognitive enhancement. Among MBSR’s core components, sitting meditation and body scan may differentially influence cognitive and emotional outcomes. Sitting meditation cultivates sustained attention to a neutral anchor—typically the breath—while simultaneously engaging open monitoring of arising thoughts, emotions, and sensations, thereby fostering meta-awareness and attentional control in accordance with contemporary classifications of mindfulness practices involving both attentional regulation and monitoring processes [27–29].

By contrast, the body scan guides attention sequentially across bodily regions to cultivate interoceptive awareness and physical relaxation, functioning as a relaxation-focused practice that emphasises sensations rather than ongoing cognitive content [27, 30, 31]. Comparing these two widely taught yet mechanistically distinct practices within the same brief protocol may help determine whether focused attention–open monitoring or interoceptive relaxation processes are more strongly linked to short-term changes in WM, mindfulness, and stress in novice healthcare students. Despite the plausibility of these distinct attentional and somatic pathways producing different outcomes, direct comparative evidence remains scarce—particularly in undergraduate healthcare students, who face uniquely high cognitive and emotional demands [32, 33]. Evidence indicates that even brief mindfulness practices lasting 8 to 10 min can improve attention, reduce mind-wandering, and enhance WM [19, 20, 34]. Four-week mindfulness protocols have also demonstrated measurable cognitive benefits and high feasibility in student populations [26]. These findings support the feasibility of using a brief yet structured intervention in this population. Accordingly, a 10-minute daily program over four weeks was selected to balance potential cognitive benefits with the practical constraints of undergraduate healthcare students.

The present study, therefore, aimed to compare the effects of two brief mindfulness practices—sitting meditation and body scan—administered for 10 min per day, five days per week, over four weeks, on WM, mindfulness, and perceived stress in undergraduate health science students.

## Methods

### Study design and setting

This study was a single-blind randomised trial conducted at a tertiary university campus in southern Thailand. The experimental protocol of the study (REC.64-436-30-2) was reviewed and approved by the Human Research Ethics Committee, Faculty of Medicine, Prince of Songkla University. The study protocol was retrospectively registered in the Thai Clinical Trials Registry No. TCTR20250916006 (https://www.thaiclinicaltrials.org/show/TCTR20250916006) on 26 August 2025.

### Participants

Study participants were undergraduate healthcare students at a tertiary university campus, aged between 18 and 25 years. Additional inclusion criteria included no history of performing sitting meditation or body scan mindfulness exercises at least four times per month within the past six months and the ability to understand and follow the study instructions, including the guided mindfulness audio instructions and the computerised WM task procedures. Participants with any condition that might affect their ability to participate in study activities, including current psychiatric symptoms, use of psychotropic medications, musculoskeletal problems, or other neurological disorders, were excluded. These criteria were applied to minimise potential confounding factors that could independently affect cognitive performance or stress responses, thereby allowing clearer interpretation of the intervention effects.

The sample size was calculated based on the primary outcome, WM performance, as measured by the Automated Operation Span Task (AOSPAN) absolute score. Estimates for the calculation were obtained from a pilot study conducted with 10 participants. The pilot data showed mean AOSPAN scores of 48.00 ± 12.17 for one condition and 60.17 ± 14.05 for the other condition. Using these parameters, the sample size was calculated to detect a difference between groups with a two-tailed significance level of α = 0.05 and 80% statistical power. The initial calculation indicated that a minimum of 22 participants per group was required. After accounting for an anticipated 20% attrition rate commonly reported in behavioural and mindfulness-based intervention studies involving student populations [35], the final target sample size was increased to 27 participants per group, resulting in a total sample size of 54 participants. Written informed consent was obtained from all eligible participants prior to study participation. The final sample consisted predominantly of female healthcare students with a mean age of approximately 21 years. Detailed demographic characteristics of the participants are presented in Table 1.

### Randomisation and blinding

Eligible participants were randomly assigned to either the sitting meditation or body scan condition using stratified randomisation by gender, religion, study major, year level, and handedness. Randomisation sequences were generated using R version 4.4.3 with a fixed block size of four to ensure balanced group allocation. The allocation sequence was prepared by a researcher who was not involved in participant recruitment, assessment, or intervention delivery, and assignments were concealed in sequentially numbered, opaque, sealed envelopes. A single-blind design was employed: outcome assessors were blinded to participants’ group allocation. Assessors had no access to the randomisation list and were not involved in delivering the intervention. Participants were informed that the study compared two validated mindfulness practices without stating hypotheses about relative effectiveness to reduce expectancy bias.

### Procedure

Researchers posted announcement posters with a QR code that directed interested students to an online screening questionnaire. The screening questionnaire was designed to collect basic participant information relevant to the study’s eligibility criteria (e.g., demographic characteristics, prior mindfulness experience, psychiatric symptoms, and use of psychotropic medications). As the purpose of the screening was limited to initial eligibility assessment rather than clinical diagnosis, the questionnaire was developed by the research team and was not based on standardized assessment instruments. All responses were self-reported by participants.

Eligible participants attended the research site for pre-assessment before initiating the assigned intervention (sitting meditation or body scan) and received instructions regarding the program. On the first day, participants were trained using a standardised expert audio clip [36]. They were instructed to practice independently at home for exactly 10 min per session, five days per week, for four weeks, guided by the same audio recording. The standardised audio recording ensured that the duration of each session was consistent across participants. After each session, participants recorded their practice experiences in a logbook. Weekly online follow-up meetings via Zoom were scheduled to monitor adherence and address concerns. After four weeks, post-intervention assessments were conducted using the same measures as at baseline.

### Intervention fidelity

Both interventions were delivered via pre-recorded audio guided by a certified mindfulness instructor in MBIs and through formal training from an accredited mindfulness centre. Weekly supervision meetings were held with the instructor to review adherence logs and address any protocol deviations. Participant logbooks were used to record daily practice, and adherence was further verified through weekly online follow-up meetings via Zoom, during which participants reported their practice completion and any difficulties encountered.

### Interventions

Sitting meditation: The participants were instructed to sit in a relaxed and alert posture on a chair, keeping their backs straight and both hands on their laps, and to close their eyes. Participants primarily focused on the breath entering and leaving the nostrils, as well as on other perceptions and maintaining a state of nonjudgmental awareness of cognitions and the stream of thoughts and distractions that pass through the mind [36].

Body scan meditation: The participants were instructed to lie on their backs with pillows under their knees and to position themselves comfortably and in a relaxed manner, placing their hands by their sides with their palms facing upward and their toes slightly apart. Participants then closed their eyes and focused their attention on each part of the body from head to feet. When they noticed their minds wandering, they were instructed to return their attention to the training [36].

### Primary outcomes measure

AOSPAN is an assessment of WM, developed by Unsworth et al. as a computer program in 2005. This computerised task is an intuitive and automated model of memory assessment that is operated using a mouse. The task includes 15 sets of trials, each consisting of two parts: a memory part (to-be-remembered element) and interference tasks. In the memory part, simple characters sized 4 × 3 (e.g., F, H, J, K, L, N, P, Q, R, S, T, and Y) are shown on the screen. Participants memorize the characters that appear in sequence, one by one, for 800 milliseconds. Then, participants recall their memories by clicking the characters in the correct order as they appeared on the screen. For the interference task, simple mathematical equations are displayed on the screen, such as (1 * 2) + 1 = ?. Participants are asked to solve the mathematical equations as quickly as possible. Then, they click the mouse to proceed to the next screen, where the answer to the equation, such as 3, is displayed. Participants are required to click the “true” or “false” box depending on their answer. All tasks include 75 letters to remember and 75 mathematical equations to solve. Higher AOSPAN scores reflect stronger WM and better academic performance, while lower scores indicate weaker WM and difficulties with attention and reasoning tasks. The test demonstrates good internal consistency (α = 0.78) and test-retest reliability (0.85) [37].

### Secondary outcomes measure

The Mindful Attention Awareness Scale (MAAS) was developed by Brown and Ryan in 2003 and translated into a Thai version in 2016 [38]. MAAS consists of 15 items rated on a six-point Likert scale, ranging from 1 (almost always) to 6 (almost never), which indicate respondents’ levels of awareness and attention to present events and experiences. High MAAS scores indicate greater mindfulness and well-being, while low scores suggest poorer mental health and less awareness. The questionnaire demonstrates good internal consistency in several samples (α = 0.80–0.87) and excellent test-retest reliability (*r* = 0.81). MAAS also shows adequate convergent validity; as expected, it correlates negatively with measures of anxiety and depression, and positively with measures of positive affect and self-esteem [39].

The Perceived Stress Scale 10 (PSS-10) measures the extent to which individuals perceive aspects of their lives as uncontrollable, unpredictable, and overloading. Participants respond to each item using a five-point Likert scale ranging from 0 (never) to 4 (very often), indicating how frequently they have experienced or thought a certain way during the past month. Total scores range from 0 to 40, with higher composite scores indicating greater perceived stress [40]. The Thai version of the PSS-10 shows good validity (*r* = 0.55–0.60), internal consistency (α = 0.80–0.84), and test-retest reliability (ICC = 0.83) for estimating perceived stress levels in a Thai population [41].

### Data analysis

**Fig. 1:**
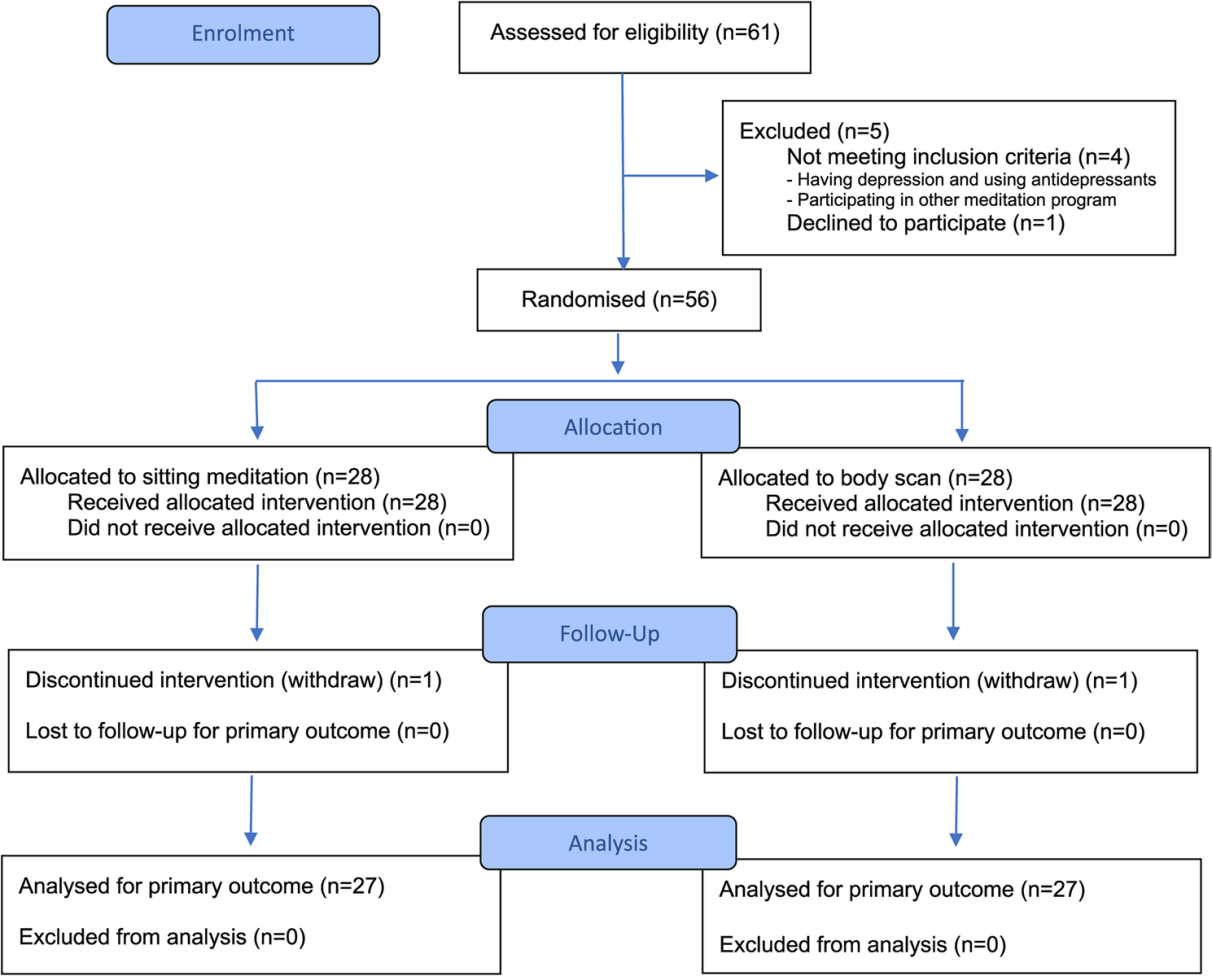
CONSORT 2025 Flow Diagram

Participants who withdrew during the intervention period and did not complete the post-intervention assessment were excluded from the final analysis. Therefore, statistical analyses were conducted using a complete-case approach including participants who completed both baseline and post-intervention measurements. In accordance with CONSORT recommendations, participant attrition and the number of analysed participants are presented in the trial flow diagram (Fig. 1). Baseline characteristics between the sitting meditation and body scan groups were compared using independent t-tests for continuous variables and Chi-square tests for categorical variables. To examine intervention effects, a two-way repeated-measures analysis of variance (RM-ANOVA) was performed to assess the main effects of time (pre- vs. post-intervention) and group (sitting meditation vs. body scan), as well as the group × time interaction for all outcome variables. Effect sizes were reported as partial eta squared (η²p). All outcome variables were analysed using parametric tests because the data were normally distributed. The level of statistical significance was set at *p* < 0.05. All statistical analyses were performed using IBM SPSS Statistics version 25.0 (IBM Corp., Armonk, NY, USA).

## Results

### Participant characteristics

Figure 1 shows the flow of participants through the trial. Sixty-one undergraduate healthcare students responded to the screening questions for the study. However, five participants (8.2%) were excluded because of depression and use of antidepressants, refusal to join the program, or participation in other meditation programs. Therefore, 56 eligible participants (91.2%) were randomly assigned to either the sitting meditation group or the body scan group. During the trial, one participant withdrew from each group, resulting in a total of 54 participants (27 per group) who completed the intervention and assessment.

### Adherence

Overall adherence to the assigned intervention programs was high. Participants in the sitting meditation group completed an average of 18.2 out of the 20 scheduled sessions (91.0%), whereas participants in the body scan group completed an average of 18.5 sessions (92.5%). Weekly follow-up via Zoom revealed no major difficulties in maintaining practice and no adverse events related to the interventions were reported.

Demographic characteristics of the participants are shown in Table 1. Most participants were female (83.3%), with a mean age of 20.96 ± 1.30 years, and had a normal body mass index (mean 21.38 ± 3.6 kg/m²). Participants in both groups were primarily Buddhist, recruited from various healthcare fields, had right arm dominance, and had no experience with any mindfulness practices. At baseline, there were no significant differences in any demographic characteristics or measured outcomes, including WM, mindfulness level, and stress scores, between the sitting meditation and body scan groups (*p* > 0.05, Table 1).

**Table 1:** Demographic characteristics of the participants

Variables	Sitting meditation group (*n* = 27)	Body scan group (*n* = 27)	*p*-value
Gender^a^			
Female	23 (85.2)	22 (81.5)	0.715
Male	4 (14.8)	5 (18.5)	
Age (years)^b^	20.89 ± 1.42 (20.33–21.45)	21.04 ± 1.19 (20.57–21.51)	0.680
BMI (kg/m^2^)^b^	20.88 ± 3.35 (19.55–22.21)	21.88 ± 3.92 (20.33–23.43)	0.319
Religion^a^			
Buddhism	22 (81.5)	20 (74.1)	0.476
Muslim	4 (14.8)	6 (22.2)	
Christian	0 (0)	1 (3.7)	
None	1 (3.7)	0 (0)	
Field of study^a^			
Medicine	4 (14.8)	3 (11.1)	0.882
Radiation technology	1 (3.7)	3 (11.1)	
Physical therapy	8 (29.6)	8 (29.6)	
Nursing	2 (7.4)	3 (11.1)	
Pharmacy	8 (29.6)	5 (18.5)	
Medicine technology	2 (7.4)	2 (7.4)	
Thai traditional medicine	2 (7.4)	2 (7.4)	
Dentistry	0 (0)	1 (3.7)	
Year^a^			
1st	1 (3.7)	1 (3.7)	0.588
2nd	7 (25.9)	5 (18.5)	
3rd	5 (18.5)	7 (25.9)	
4th	12 (44.4)	14 (51.9)	
5th	2 (7.4)	0 (0)	
6th	0 (0)	0 (0)	
Dominant hand^a^			
Right	23 (85.2)	26 (96.3)	0.159
Left	4 (14.8)	1 (3.7)	
Exercise activity in the past 6 months^a^			
Yes	22 (81.5)	22 (74.1)	0.513
No	5 (18.5)	7 (25.9)	
Level of stress^a^			
Low	0 (0)	2 (7.4)	0.352
Moderate	23 (88.9)	22 (81.5)	
High	3 (11.1)	3 (11.1)	
AOSPAN^b^			
Absolute score	46.85 ± 12.65 (41.85–51.86)	43.52 ± 11.81 (38.85–48.19)	0.322
Total correct score	61.37 ± 8.57 (57.98–64.76)	59.70 ± 8.25 (56.44–62.97)	0.470
MAAS^b^	61.93 ± 8.75 (58.47–65.39)	61.74 ± 8.69 (58.31–65.18)	0.938
T-PSS-10^b^	20.26 ± 3.06 (19.05–21.47)	20.78 ± 3.23 (19.50–22.05)	0.547

Abbreviations: *BMI *body mass index, *AOSPAN *Automated Operation Span Task, *MAAS *Mindfulness Attention Awareness Scale, *T-PSS-10 *Thai-Perceived Stress Scale

^a^ The data are presented by n (%), and *p*-values were assessed by the Chi-square test

^b^ The data are presented by mean ± SD (95% confidence interval), and p-values were assessed by the independent t-test

### Outcomes

Changes in the primary and secondary outcomes are presented in Table 2. For WM, a significant main effect of time was observed for both AOSPAN absolute scores (F = 27.621, *p* < 0.001, η²*p* = 0.347) and total correct scores (F = 26.698, *p* < 0.001, η²*p* = 0.339), indicating large effects of time across both groups. However, no significant group × time interaction was found for either outcome (AOSPAN absolute score: *p* = 0.365; AOSPAN total correct score: *p* = 0.445), suggesting that the magnitude of change did not differ between the sitting meditation and body scan groups.

**Table 2:** Effects of sitting meditation and body scan on WM, mindfulness, and perceived stress

Group and outcomes	Pre-intervention (T0)	Post-intervention (T1)	Mean difference (T1 – T0)	Time effect F (*p*), η²*p*	Group × time F (*p*), η²*p*
AOSPAN absolute score					
Sitting meditation	46.85 ± 12.65 (41.85, 51.86)	54.85 ± 11.90 (50.15, 59.56)	8.00 ± 11.94 (3.28, 12.72)	27.621 (< 0.001), 0.347	0.836 (0.365), 0.016
Body scan	43.52 ± 11.81 (38.85, 48.19)	54.86 ± 13.58 (49.52, 60.26)	11.37 ± 14.98 (5.45, 17.30)		
AOSPAN total correct					
Sitting meditation	61.37 ± 8.57 (57.98, 64.76)	66.56 ± 6.87 (63.84, 69.27)	5.19 ± 8.93 (1.65, 8.72)	26.698 (< 0.001), 0.339	0.592 (0.445), 0.011
Body scan	59.70 ± 8.25 (56.44, 62.97)	66.70 ± 7.03 (63.92, 69.49)	7.00 ± 8.39 (3.68, 10.32)		
MAAS					
Sitting meditation	61.93 ± 8.75 (58.47, 65.39)	66.67 ± 8.32 (63.38, 69.96)	4.74 ± 5.90 (2.41, 7.07)	38.623 (< 0.001), 0.420	1.281 (0.263), 0.024
Body scan	61.74 ± 8.69 (58.31, 65.18)	68.59 ± 5.28 (66.50, 70.68)	6.85 ± 7.69 (3.81, 9.89)		
T-PSS-10					
Sitting meditation	20.26 ± 3.06 (19.05, 21.47)	19.85 ± 2.76 (18.76, 20.94)	-0.41 ± 3.18 (-1.66, 0.85)	4.750 (0.034), 0.084	1.536 (0.221), 0.029
Body scan	20.78 ± 3.23 (19.50, 22.05)	19.30 ± 2.96 (18.13, 20.47)	-1.48 ± 3.19 (-2.73, -0.22)		

Abbreviations: *AOSPAN *Automated Operation Span Task, *MAAS *Mindfulness Attention Awareness Scale, *T-PSS-10 *Thai-Perceived Stress Scale

Data are presented as mean ± standard deviation (95% confidence interval). Time and group × time interaction effects were analysed using two-way repeated-measures ANOVA

For mindfulness, MAAS scores showed a significant main effect of time (F = 38.623, *p* < 0.001, η²*p* = 0.420), indicating a large effect. The group × time interaction was not statistically significant (*p* = 0.263, η²*p* = 0.024), indicating comparable changes between the two groups.

For perceived stress measured using the T-PSS-10, scores changed slightly over time (F = 4.750, *p* = 0.034, η²*p* = 0.084); however, no significant group × time interaction was observed (*p* = 0.221, η²*p* = 0.029), indicating no significant difference between the sitting meditation and body scan groups.

Effect sizes are reported as partial eta squared (η²p). The magnitude of effect sizes was interpreted as small (η²*p* = 0.01), medium (η²*p* = 0.06), and large (η²*p* = 0.14).

## Discussion

This study aimed to compare the effects of sitting meditation and body scan practices on WM as the primary outcome, as well as mindfulness level and stress as secondary outcomes, among healthcare undergraduate students. The findings showed that both sitting meditation and body scan interventions were associated with significant changes in WM over time. No statistically significant differences were observed between the two groups. Similarly, mindfulness scores increased significantly in both groups. Although stress scores changed slightly over time, no significant between-group difference was identified. Therefore, the present findings do not support a clear advantage of either sitting meditation or body scan for stress reduction.

Both mindfulness practices were associated with changes in WM, which is consistent with previous studies suggesting that mindfulness training may be linked to improvements in cognitive functions. For example, Youngs et al. [42] found that even a single brief mindfulness meditation session significantly enhanced visual short-term memory, while Quach et al. [26] showed that a mindfulness meditation intervention significantly improved WM capacity in adolescents compared to a Hatha yoga and waitlist control group. Additionally, Fischer et al. [43] showed that an eight-week body scan intervention significantly improved interoceptive accuracy and confidence in bodily perception, suggesting that focused attention on bodily sensations can enhance cognitive and self-awareness processes. Mindfulness practices are believed to strengthen attentional control, enhance executive functioning, and reduce cognitive load, all of which are important for WM performance. Therefore, the changes observed in both the sitting meditation and body scan groups in this study may be explained by these mechanisms, as supported by prior research.

Similarly, the changes in mindfulness scores observed in both groups can be attributed to the fundamental nature of sitting meditation and body scan practices, which cultivate mindful awareness by encouraging sustained attention to present-moment experiences in a nonjudgmental manner. This finding is consistent with previous studies, which show that even brief mindfulness interventions may be associated with increased mindfulness. For example, Mrazek et al. [19] reported that a two-week mindfulness training program enhanced mindfulness and reduced mind-wandering among undergraduate students, and Tang et al. [34] found that five days of integrative body–mind training significantly improved self-reported mindfulness and attentional control. Moreover, the structured and systematic nature of both practices in the present study likely provided participants with repeated opportunities to regulate attention, which is a core component of mindfulness development [44].

Although the body scan group showed somewhat larger changes in working memory and mindfulness, these differences were not statistically significant between groups. Therefore, these findings should be interpreted with caution. The observed pattern may reflect differences in attentional engagement and interoceptive awareness associated with body scan practice [34, 43]; however, such explanations remain speculative and cannot be confirmed based on the present findings. Furthermore, given the absence of statistically significant between-group differences, these interpretations should not be considered evidence of greater effectiveness of one intervention over the other. It is possible that mindfulness practices, including body scan, are associated with changes in neural regions involved in attention, self-awareness, and memory, such as the anterior cingulate cortex, insula, and hippocampus [45]. However, given that both interventions are grounded in similar mindfulness principles, such mechanisms may not be specific to one practice and cannot explain differences between groups in the current study.

Stress scores changed slightly over time in both groups; however, these findings should be interpreted cautiously given the small magnitude of change and the absence of significant between-group differences. One possible explanation is that mindfulness-based practices, including sitting meditation and body scan, may promote greater awareness of internal experiences and emotional states through focused attention and non-judgmental observation. Previous neuroimaging studies have suggested that mindfulness practices are associated with structural and functional changes in brain regions involved in emotion regulation, including the hippocampus, anterior cingulate cortex, and medial prefrontal cortex [45]. These mechanisms may contribute to changes in perceived stress over time. However, the relatively small magnitude of change observed in the present study may reflect the short duration and low intensity of the intervention. This interpretation is consistent with findings reported by Khoury et al. [46], who reported that while MBIs such as MBSR have been associated with reductions in perceived stress, the magnitude of the effect often depends on the length and intensity of the training. Therefore, further studies with longer intervention periods, greater training intensity, and appropriate control groups are needed to clarify the potential effects of mindfulness practices on stress outcomes.

The absence of significant between-group differences in both primary and secondary outcomes may be explained by the inherent similarity in the effects of the two mindfulness practices, sitting meditation and body scan. Both interventions are grounded in the principles of mindfulness and aim to cultivate present-moment awareness, attentional regulation, and nonjudgmental observation of internal experiences. Sitting meditation typically emphasises focused attention on the breath, whereas body scan guides attention sequentially through bodily sensations. However, both practices share the overarching aim of enhancing self-awareness and reducing psychological distress. Given these shared therapeutic mechanisms, both practices may contribute to similar patterns of change in cognitive and emotional outcomes, leading to no statistically significant differences between the groups. Thus, even though sitting meditation emphasises focused attention and open monitoring, whereas the body scan targets interoceptive, relaxation-focused attention, their shared cultivation of sustained present-moment awareness may account for the absence of statistically significant between-group differences in this trial.

### Limitations

Several factors should be considered when interpreting these findings. First, the screening questionnaire was developed by the research team to collect basic eligibility-related information and was not based on standardized assessment tools. Although this approach was appropriate for preliminary screening, reliance on self-reported and non-validated measures may have introduced misclassification bias, potentially influencing participant selection and limiting internal validity. Second, the sample consisted predominantly of female healthcare students (83%), and the study was conducted at a single university, which may limit the generalisability of the results to more gender-balanced populations and other educational settings. Third, participants with current psychiatric symptoms, psychotropic medication use, neurological disorders, or musculoskeletal problems were excluded to minimise potential confounding influences on cognitive performance and ensure safe participation in the intervention. While these criteria strengthened internal validity, they may limit the applicability of the findings to students experiencing higher levels of psychological distress, who may benefit most from mindfulness-based practices. Fourth, because the AOSPAN was administered at both baseline and post-intervention, improvements in WM performance may partly reflect practice effects associated with repeated testing. Furthermore, the relatively brief intervention period, reliance on self-reported psychological measures, and lack of follow-up assessment may have limited the ability to detect longer-term or more nuanced effects of the interventions. Nevertheless, the results provide valuable preliminary evidence and a foundation for future studies involving larger and more diverse samples, multi-site designs, longer intervention periods, objective cognitive measures, and longitudinal follow-up to better understand the sustainability and mechanisms of change associated with brief mindfulness practices.

Finally, multiple outcome measures were analysed without formal adjustment for multiple comparisons, which may increase the risk of Type I error whereby some statistically significant findings could occur by chance. Consequently, the results, particularly those related to secondary outcomes, should be interpreted with caution. In addition, the study was retrospectively registered in a clinical trial registry. Although ethical approval was obtained prior to participant recruitment, the lack of prospective registration may reduce transparency regarding the pre-specification of study outcomes and increase the potential risk of selective reporting bias. These considerations should be taken into account when interpreting the findings.

## Conclusion

Both sitting meditation and body scan were associated with changes in working memory and mindfulness over time among healthcare students. Stress scores also changed slightly over time, although no significant between-group differences were observed for any outcome. These findings suggest that both mindfulness-based practices may be feasible approaches for healthcare students who are new to mindfulness practice. However, neither sitting meditation nor body scan demonstrated statistical superiority when directly compared in the present study.

### Practical implications

These findings suggest that brief mindfulness-based practices, such as sitting meditation and body scan, may be feasible approaches for inclusion in healthcare education settings. Structured mindfulness activities delivered during lecture breaks, laboratory sessions, or through online platforms may be associated with changes in working memory, mindfulness, and perceived stress among healthcare students. Flexible delivery formats, including workshops and mobile-guided practice, may also encourage student engagement and accessibility. However, further studies with longer follow-up periods and appropriate control groups are needed to clarify the long-term effects of these practices.

## Data Availability

Data produced and/or analysed during this study are available from the corresponding author on reasonable request.

## Abbreviations

WM: Working memory
MBIs: Mindfulness-based interventions
MBSR: Mindfulness-based stress reduction
MBCT: Mindfulness-based cognitive therapy
AOSPAN: Automated Operation Span Task
MAAS: Mindful Attention Awareness Scale
